# Fermentation Process Evaluation of a Sustainable and Innovative Miso Made from Alternative Legumes

**DOI:** 10.3390/foods14234131

**Published:** 2025-12-02

**Authors:** Rafaela Santos, Beatriz Parente, Mariana Mota, Anabela Raymundo, Catarina Prista

**Affiliations:** LEAF—Linking Landscape, Environment, Agriculture and Food Research Center, Associated Laboratory TERRA, Instituto Superior de Agronomia, University of Lisbon, Tapada da Ajuda, 1349-017 Lisbon, Portugal; rcsantos@isa.ulisboa.pt (R.S.); biaparente@gmail.com (B.P.); mariana@isa.ulisboa.pt (M.M.); anabraymundo@isa.ulisboa.pt (A.R.)

**Keywords:** miso, fermentation, chickpea, lupin, cowpea, salt reduction, sustainable and innovative food

## Abstract

Miso is a traditional Japanese seasoning produced by fermenting soybean. However, in Portugal, most soybean is imported. This study focused on producing sustainable and innovative misos using legumes traditionally consumed in Portugal—chickpea, lupin, and cowpea—and assessing their fermentation. Each legume was blended with 3% or 12% NaCl and inoculated with a selected microbial consortium comprising *Aspergillus oryzae* (koji), *Debaryomyces hansenii*, *Zygosaccharomyces rouxii*, *Candida versatilis*, *Lactiplantibacillus plantarum*, *Leuconostoc mesenteroides*, and *Tetragenococcus halophilus*. Fermentation was carried out at 20 ± 1 °C for 18 months. During this period, microbial viability, pH, total soluble solids, soluble protein, phenolic compounds, reducing sugars, and organic acids were monitored. Soybean misos were also produced and analysed at the beginning and end of fermentation for comparison. Chickpea misos showed the highest accumulation of soluble protein, phenolic compounds, reducing sugars, and organic acids. In contrast, cowpea misos exhibited the lowest levels of these soluble fractions. Lupin misos displayed the most pronounced salt-dependent behaviour. Compared to the alternative legume-based misos, soybean misos did not exhibit distinct final characteristics. These findings highlight the potential of these legumes as alternative substrates for miso production, supporting the development of sustainable, innovative, lower-salt foods with strong cultural and regional relevance.

## 1. Introduction

Fermentation is one of the oldest food processing methods, believed to have originated around 8000 BC during the rise of early civilisations. In ancient cultures, including the Roman Empire, fermented milk products were already used for therapeutic purposes, such as treating gastroenteritis [1]. This biochemical process relies on the metabolic activity of microorganisms—typically bacteria, yeasts, and filamentous fungi—and can occur either spontaneously or in a controlled environment [2]. Fermented foods are common across various cultures and include dairy products, meat and fish, beverages, vegetables, legumes, and cereals. Among them, the fermentation of legumes has been associated with improvements in their nutritional properties, promoting an increase in health-promoting compounds and a reduction in antinutritional factors [1,3]. However, many legume-based fermented foods, such as doubanjiang, soy sauce and miso, are traditionally produced with high salt levels and are recognised as contributors to dietary salt intake [4]. In this context, salt reduction is now recognised as a key public health strategy to lessen the burden of non-communicable diseases, leading consumers to increasingly seek lower-salt versions of fermented products that still retain their characteristic flavour and benefits [4].

Miso is one of the most important traditional foods in the Japanese diet. It is a semi-solid seasoning paste produced by fermenting soybeans [2,3]. Miso production involves a two-step fermentation process. First, a substrate (usually steamed rice or barley, or cooked soybeans) is inoculated with the mould *Aspergillus oryzae* to produce koji. The koji is then mixed with cooked and mashed soybeans, salt at a concentration between 5% and 13%, and water. This mixture is left to ferment further through the metabolic activity of yeasts and bacteria [2,5,6]. However, miso is often still produced with native yeasts and bacteria and under spontaneous and uncontrolled conditions, which can compromise its quality and safety. The use of specific, well-characterised microorganisms has been shown to enhance the fermentation process [2], contributing to the development of desirable organoleptic properties—including flavour, aroma, texture, and colour—and to improvement of the nutritional profile and probiotic potential of the final product [2,5]. Furthermore, the standardisation of miso production is essential for ensuring greater consistency in product quality and traceability—crucial factors for the food industry [7].

As previously described, koji plays a key role in miso production due to the enzymatic activity of *A. oryzae*, which secretes hydrolytic enzymes, particularly amylases and proteases [2,5]. Amylases break down polysaccharides, such as starch, into glucose and oligosaccharides, providing fermentative yeasts with available sugars. Proteases break down proteins into peptides and free amino acids, improving digestibility [2,5,8,9]. These low molecular weight compounds serve as key substrates for the microbial consortium involved in miso fermentation. Salt-tolerant yeasts, such as *Zygosaccharomyces rouxii*, ferment reducing sugars into ethanol and aroma-active compounds, which are crucial in shaping the sensorial characteristics of miso [2,8]. In contrast, salt-tolerant lactic acid bacteria (LAB), like *Tetragenococcus halophilus*, utilise both reducing sugars and peptides to produce lactic acid, promoting acidification, enhancing flavour complexity, and improving microbial stability. LAB also enhance miso’s nutritional quality by breaking down peptides and lipids, releasing bioactive compounds such as free amino acids and short-chain fatty acids [2,9].

Although soybean (*Glycine max*) is the primary protein source in miso, its use in Portugal is limited due to very low local production of soybean and, consequently, a high dependence on imported raw material [10]. Meanwhile, chickpea (*Cicer arietinum* L.), lupin (*Lupinus* spp.), and cowpea (*Vigna unguiculata* L. Walp) are widely cultivated across the country and form an integral part of the national food culture [11].

These legumes, traditionally consumed in Portugal, present a diverse nutritional profile once cooked, with characteristics that are often comparable—and, at times, superior—to those of soybean (Table 1). Soybean is widely recognised for its high content of protein and fibre and is also an important source of thiamine (vitamin B1), folate (vitamin B9), potassium, phosphorus, magnesium, and iron. However, chickpea, lupin, and cowpea also exhibit relevant nutritional qualities. They are sources of both protein and fibre, with lupin standing out for its particularly high protein content, and chickpea for its richness in fibre. Chickpea and cowpea also contain starch—a relevant component in fermentation processes. In terms of vitamins, these legumes are notable sources of folate, with cowpea showing the highest content and also providing thiamine. Regarding minerals, both lupin and cowpea display appreciable levels of phosphorus, with the former also contributing iron and the latter potassium. Chickpea, while having a more modest mineral profile, still constitutes a source of iron [12,13,14,15,16].

Despite the nutritional value and cultural significance of these alternative legumes, they remain underutilised in the modern food sector, particularly in the development of innovative, attractive, and value-added products. Fermenting chickpea, lupin, and cowpea could help to increase their consumption, support agrobiodiversity, promote the circular use of resources, and ultimately contribute to the creation of sustainable and innovative fermented foods with a strong regional connection [18].

The main objective of this work was to produce sustainable and innovative misos using legumes widespread in Portugal to replace the traditional use of soybean, while also reducing the concentration of salt. Additionally, the study aimed to evaluate the long-term fermentation process to understand its impacts on microbiology, physicochemical properties, substrate consumption, and metabolite production.

Eight types of miso were produced using chickpea, lupin, cowpea, and soybean, with two salt concentrations (3% or 12% NaCl) and inoculated with a defined microbial consortium (one filamentous fungus, three yeast strains, and three LAB strains). The fermentation process was periodically monitored by assessing microbial viability, pH, total soluble solids (TSS), concentrations of reducing sugars and organic acids, as well as levels of soluble protein and phenolic compounds.

Traditional soybean misos, produced under the same conditions, were analysed at the beginning and at the end of the fermentation process. These time points were assessed to understand the overall impact of new legumes on the fermentation products, with the primary objective of comparing the final characteristics of the soybean misos with those of the six innovative misos. The alternative legume-based misos were developed to support dietary diversification and promote the consumption of locally grown legumes.

## 2. Materials and Methods

### 2.1. Microorganisms and Culture Conditions

*Aspergillus oryzae* was obtained from Vision Brewing (Nedlands, Australia). *Debaryomyces hansenii* JCM 2162, *Zygosaccharomyces rouxii* JCM 22060 and *Tetragenococcus halophilus* subsp. *halophilus* JCM 5888 were purchased from the Microbe Division JCM (Tsukuba, Japan). *Candida versatilis* ISA 1983 was sourced from the ISA Yeast Strain Library. *Lactiplantibacillus plantarum* subsp. *plantarum* DSM 20205 and *Leuconostoc mesenteroides* subsp. *mesenteroides* DSM 20343 were purchased from the Leibniz Institute DSMZ (Braunschweig, Germany). All strains belong to microbial species commonly report in miso fermentations and were originally isolated from this fermented food [2,19,20].

*A. oryzae* was cultivated on Potato Dextrose Agar (PDA; Merck, Darmstadt, Germany) at 20 ± 1 °C until vigorous growth was observed. The yeasts were cultivated in Yeast Peptone Dextrose (YPD; Merck, Darmstadt, Germany) broth at 28 °C under agitation (130 rpm), while the lactic acid bacteria (LAB) were grown in Man, Rogosa and Sharpe (MRS; Merck, Darmstadt, Germany) broth at 28 °C without agitation. The medium for *C. versatilis* and *T. halophilus* was supplemented with 5% NaCl, and *T. halophilus* was incubated at 30 °C without agitation. All cultures were incubated until vigorous growth was observed.

### 2.2. Koji Production

A Portuguese Japonica variety of *Oryza sativa* L., Blanched Carolino Rice (Golden Sun, Portugal), was washed and soaked overnight, approximately 14 h, at 20 ± 1 °C. After draining the water, the rice was steam-cooked at 100 °C for 30 min using a Thermomix TM31 (Vorwerk, Wuppertal, Germany) and then allowed to cool at 20 ± 1 °C. The cooled rice was distributed in a perforated aluminium tray covered with cheesecloth and inoculated with a spore suspension of *A. oryzae* (1 × 10^4^ spores/g of koji). After mixing the inoculum with the rice, the tray was covered with cheesecloth and incubated at 20 ± 1 °C until fungal growth became visible around the rice grains. During incubation, the rice (koji) was sprayed with demineralised water and manually revolved three times per day.

### 2.3. Legumes Used for Miso Production

Four standard commercial organic dried legumes—chickpea (Casal Vouga cultivar, Seara, Portugal), lupin (Estoril cultivar, Agrinemus, Portugal), cowpea (Fradel cultivar, local Portuguese farmer) and soybean (JS-335 cultivar Próvida, Portugal)—were purchased from local retail stores. The legumes were washed and soaked overnight at 20 ± 1 °C. In the case of lupin, a 10-day soaking process was carried out prior to use, during which the water was replaced twice daily to reduce the alkaloid content. After draining, the legumes were cooked separately in a Labo Autoclave MLS-3020U (Panasonic, Hamburg, Germany) at 120 °C and 0.1 MPa for 20 min. A smooth paste was then prepared by grinding each legume using a Quad Blade Chopper CH580 (Kenwood, Havant, UK).

### 2.4. Miso Production

Six types of miso were produced using three different legumes (chickpea, lupin, and cowpea), each combined with two concentrations of sea salt (3% or 12%, *w*/*w*), purchased from a local supermarket. Miso production followed two distinct formulations.

The 3% salt formulation contained 72.9 g legume, 20.9 g koji, 3 g sea salt, 2.2 g non-pasteurised miso, and 1 g inoculum (yeasts and LAB) per 100 g final product. The 12% salt formulation consisted of 65.1 g legume, 20.9 g koji, 12 g sea salt, 1 g non-pasteurised miso, and 1 g inoculum. Yeasts and LAB, previously cultured as described in Section 2.1, were concentrated by centrifugation, resuspended in water and inoculated at final cell densities of 1 × 10^4^ cells/g and 1 × 10^6^ cells/g of miso, respectively.

The raw materials were mixed until a uniform pasty consistency was achieved. The resulting paste was firmly packed into cylindrical glass jars (140 mL capacity; Ø 7 × 5.5 cm) pre-coated with salt on the inner walls and bottom. Before sealing, the surface of the paste was covered with a layer of salt. Fermentation was carried out at 20 ± 1 °C for 18 months. Two jars of each type of miso were collected at regular intervals to monitor the fermentation process.

Soybean misos with 3% or 12% NaCl were also produced, following the same procedure described for the six innovative misos, and analysed at the initial (T0) and final (T18) fermentation time points.

To ensure clarity throughout the Section 3 (Results and Discussion) the following abbreviations were adopted: CM3 and CM12 for chickpea-based misos (containing 3% and 12% NaCl, respectively); LM3 and LM12 for lupin-based misos (3% and 12% NaCl); CoM3 and CoM12 for cowpea-based misos (3% and 12% NaCl); and SM3 and SM12 for soybean-based misos (3% and 12% NaCl). Fermentation time points were also standardised as follows: T0 (start of fermentation), T3 (3 months), T6 (6 months), T12 (12 months), and T18 (18 months or end of fermentation). An exception applies to Section 3.1, where shorter time intervals were used to assess microbial viability.

### 2.5. Microbiological Analysis

The cell viability of yeasts and LAB in miso was assessed initially and throughout the fermentation process. For each sample, serial dilutions ranging from 10^−1^ to 10^−6^, were prepared in demineralised water. Three dilutions were then inoculated in duplicate onto YPD medium supplemented with 25 mg/L of chloramphenicol, and onto MRS medium supplemented with 20 mg/L of cycloheximide (Merck, Darmstadt, Germany). The plates were incubated at 20 ± 1 °C for 5 to 9 days. The resulting colonies were counted and expressed as the log_10_ of colony-forming units (CFU) per gram of fresh biomass.

### 2.6. Determination of pH and Total Soluble Solids (TSS)

The pH values of miso were measured directly on its surface using a Lab pH Meter pHM92 (Radiometer, Copenhagen, Denmark), equipped with a pH-Elektrode BlueLine 21 pH (SI Analytics, Weilheim, Germany). The total soluble solids (TSS) content was determined by diluting miso samples (1:10, *w*/*v*) in demineralised water, followed by centrifugation at 3150× *g* for 10 min at 4 °C using a Centrifuge 5810 R (Eppendorf, Hamburg, Germany). After centrifugation, an aliquot of the supernatant was transferred to a HI 06801 Refractometer (Hanna Instruments, Smithfield, RI, USA) for the quantification of TSS, expressed in °Brix. Each parameter was assessed at least three times.

### 2.7. Soluble Protein Content

Soluble protein was determined using the Bradford method [21]. Miso samples were diluted 1:10 (*w*/*v*) in demineralised water, vigorously stirred, and centrifuged at 3150× *g* for 10 min at 4 °C. An aliquot (500 µL) of the supernatant was mixed with 500 µL of Bradford reagent (Merck, Darmstadt, Germany) and incubated at 20 ± 1 °C for 1 min. Absorbance was measured at 595 nm using a Libra S22 UV/VIS Spectrophotometer (Biochrom, Cambridge, UK). Bovine serum albumin (Merck, Darmstadt, Germany) was used to prepare the standard curve, from which protein concentration was calculated and expressed as milligrams per 100 g of fresh biomass. Each sample was analysed in triplicate.

### 2.8. Soluble Phenolic Compounds

Soluble phenolic content was assessed spectrophotometrically using 1 mL of the supernatant obtained after centrifugation, as described in Section 2.7. Absorbance was measured at 280 nm using a quartz cuvette [22]. Results were expressed as milligrams of gallic acid equivalents (GAE; Merck, Darmstadt, Germany) per 100 g of fresh biomass, based on a standard calibration curve prepared with gallic acid. Miso samples were analysed in triplicate.

### 2.9. Reducing Sugars and Organic Acids by High-Performance Liquid Chromatography (HPLC)

Miso samples were diluted 1:10 (*w*/*v*) in 50 mM H_2_SO_4_ (Merck, Darmstadt, Germany), vigorously stirred, and centrifuged at 3150× *g* for 10 min at 4 °C. The supernatant was diluted (1:2, *v*/*v*) with the same acid solution and filtered through a 0.22 μm pore-size membrane. The samples were analysed using a Chromaster HPLC System (Hitachi, Tokyo, Japan) equipped with a Rezex™ ROA Organic Acid H+ (8%) ionic exclusion column (Phenomenex, Torrance, CA, USA) at 65 °C. Detection was carried out using a UV-VIS detector (5420) for organic acids and a refractive index detector (5450) for sugars, glycerol, and ethanol. A volume of 20 μL from each sample was injected in triplicate, and 5 mM H_2_SO_4_ was used as the mobile phase at a flow rate of 0.5 mL/min.

### 2.10. Statistical Analysis

The mean and standard deviation of the experimental data were calculated using R (version 4.4.2; R Foundation for Statistical Computing, Vienna, Austria). A three-way analysis of variance (ANOVA) was performed to assess the effects of the factors. However, as the assumptions of normality and homogeneity of variances were violated, the non-parametric Kruskal–Wallis test was then applied. The independent variables were treated as factors for the Kruskal–Wallis test. Differences between the groups were tested for statistical significance at α = 0.05. All statistical analyses were carried out in R using the *stats* and *agricolae* packages.

## 3. Results and Discussion

### 3.1. Viability of Yeasts and Lactic Acid Bacteria (LAB)

Viable and culturable populations of yeasts and LAB were detected during the early stages of fermentation (Table 2). LAB tended to persist longer than yeasts: all miso types maintained culturable yeasts up to day 30 and LAB up to day 60. In chickpea-based misos and CoM12, yeasts declined more rapidly, becoming undetectable by day 60, while LAB were no longer culturable by day 90 in CM12 and LM3. CoM3 was the only miso to sustain viable yeasts and LAB until day 180, highlighting its distinct microbial profile. By day 270, however, no culturable microorganisms were detected in any miso.

Nonetheless, the absence of culturable cells does not necessarily indicate that all microbial activity has ceased. Various stress conditions, including physical and chemical factors and microbial competition, are known to create suboptimal conditions for microbial growth, thereby inducing a viable but non-culturable (VBNC) state in yeast and bacterial cells [23]. In non-spore-forming bacteria, the VBNC state has been described as a major adaptive response to adverse external stresses [24]. Cells in this state remain viable yet can no longer be cultured on conventional media and lose their ability to form colonies, although they may persist for long periods [24].

Within the miso microenvironment, multiple stress factors coexist, including high salt concentrations, progressive acidification due to the accumulation of organic acids throughout fermentation, and a competitive microbiota composed of yeasts and LAB [2,19,20]. This combination of factors is consistent with the decline in culturable cells observed after 60 days (Table 2) and suggests that part of the community may have entered a VBNC state rather than being completely inactivated. VBNC cells may still sustain low-level metabolic activity, which, together with residual enzymatic activity from the koji, could continue to modulate the chemical environment of the miso, particularly during maturation [25].

### 3.2. Evaluation of pH and Total Soluble Solids (TSS)

**Figure 1:**
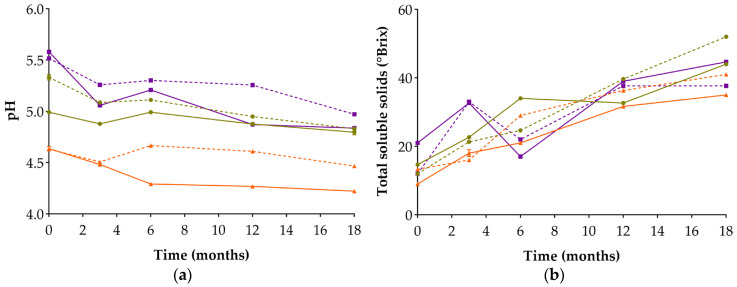
Evolution of pH values (**a**) and variation in total soluble solids (**b**) in the six alternative legume-based misos over 18 months of fermentation. Values are expressed as mean ± standard deviation (*n* = 3). Circles: chickpea-based misos; triangles: lupin-based misos; squares: cowpea-based misos. Solid lines: 3% NaCl; dashed lines: 12% NaCl.

The pH values shown in Figure 1 indicate that chickpea misos reached final pH values close to 5.0, similar to those reported in the literature for traditional miso [20,26]. LM3 and LM12 consistently exhibited the lowest pH from the beginning of the fermentation, whereas CoM12 showed the highest pH overall. Miso produced with 12% NaCl consistently showed higher pH values than those with 3% NaCl over fermentation, in agreement with previous studies reporting that lower salt levels in fermented foods generally result in lower pH [27,28]. Throughout fermentation, pH generally decreased, with a more pronounced decline during the early stages, when the highest viable and culturable counts were observed (Table 2). In line with this overall trend, CoM3 exhibited the greatest reduction in pH among all misos.

The high LAB counts observed in CoM3 on day 30, together with the presence of heterofermentative species such as *L. mesenteroides* and *T. halophilus*—both known to produce lactic and acetic acids [29,30]—may have contributed to the more marked acidification observed in this miso. In contrast, LM3 showed a more gradual decline in pH over the first six months, which may be linked to early-stage microbial dynamics. LM3 exhibited higher counts of yeasts and LAB at days 15 and 30 than LM12, suggesting a more intense early fermentative phase and a progressive accumulation of fermentative acids in low salt misos, that, within the buffered miso matrix, likely contributed to the gradual decrease in pH.

Regarding TSS, an overall increase was observed in all misos (Figure 1). CoM3 started with the highest initial value and, despite fluctuations, ended with one of the highest final concentrations; the only higher value was observed for CM12, which exhibited the greatest relative increase (333.3%), reaching 52 °Brix. Notably, CoM3 was also the only miso in which TSS evolution inversely mirrored pH. Cooked cowpea contains more starch than chickpea and less total fibre than lupin [13,14,15,16], which may have contributed to the higher initial availability of soluble compounds. Furthermore, the enzymatic hydrolysis of complex macromolecules likely promoted the production of low molecular weight solutes, such as reducing sugars and organic acids [31], contributing to both the elevated TSS and the atypical pH trend observed in CoM3.

By contrast, LM3 tended to exhibit lower TSS values and ended with the lowest final concentration (35 °Brix). This result may be associated with its persistently lower pH, which could have limited enzyme activity, since most enzymes from *A. oryzae* (including proteases and amylases) display optimal activity under slightly acidic conditions (typically between pH 5.5 and 6.0) [32]. Additionally, the low starch content and high protein concentration of cooked lupin may limit substrate availability and hinder solute diffusion [33], contributing to the lower TSS values observed in lupin miso. These effects were less pronounced in LM12, suggesting that salt concentration influenced solute mobilisation in lupin-based misos.

### 3.3. Soluble Protein and Soluble Phenolic Content

**Figure 2:**
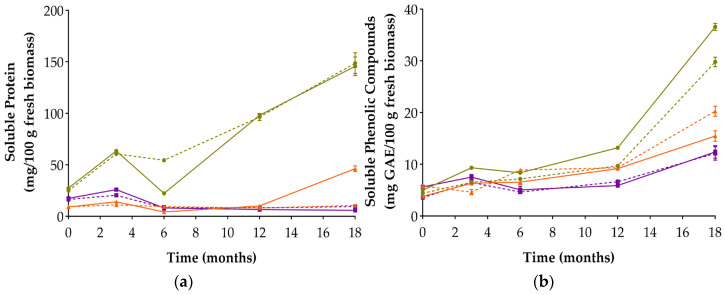
Soluble protein content (**a**) and soluble phenolic compounds (**b**) in the six alternative legume-based misos over 18 months of fermentation. Values are expressed as mean ± standard deviation (*n* = 3). Circles: chickpea-based misos; triangles: lupin-based misos; squares: cowpea-based misos. Solid lines: 3% NaCl; dashed lines: 12% NaCl.

Chickpea-based misos clearly stood out for their high concentrations of soluble protein and soluble phenolic compounds, showing the greatest increases throughout fermentation (Figure 2). CM3 and CM12 reached the highest final values: 145.799 and 148.690 mg/100 g of soluble protein, and 36.572 and 33.382 mg GAE/100 g of soluble phenolic compounds, respectively. This behaviour may reflect sustained enzymatic activity, potentially favoured by the nutritional composition of chickpea [34,35]. Among the three legumes, cooked chickpea exhibited moderate starch and fibre levels, which—in combination with their overall nutritional profile—may have contributed to increased enzymatic accessibility and solute diffusion during fermentation [13,14,15,16,36]. Chickpea also contained the highest vitamin B6 (pyridoxine) levels—a cofactor in amino acid metabolism—and moderate levels of calcium and magnesium, which might have supported protein solubility by limiting aggregation or precipitation [13,37,38,39,40]. This result is consistent with the marked increase in TSS observed in CM12 (see Section 3.2), suggesting a close link between solute accumulation and matrix composition.

Cowpea-based misos, in contrast, exhibited the lowest concentrations of soluble protein and phenolic compounds from month 6 onwards and were the only misos to show a net decrease in protein between T0 and T18. Although cowpea had a slightly higher total protein content than chickpea, this was not reflected in solubilisation. Compositional factors, such as its higher starch and lower fibre content, and a mineral profile characterised by elevated phosphorus and potassium, may have provided limited support for enzymatic hydrolysis, while possible interactions between proteins and phenolics may have further restricted solute release [13,14,15,41,42,43].

Although the effect of salt concentration on phenolic retention was not consistent across legumes, the differences observed between CM3/CM12 and LM3/LM12 suggest that matrix-specific properties played a key role in modulating NaCl’s influence on solute release. This result highlights the complexity of interactions between salt, legume type, and fermentation dynamics.

### 3.4. Reducing Sugars and Organic Acids Evolution

**Figure 3:**
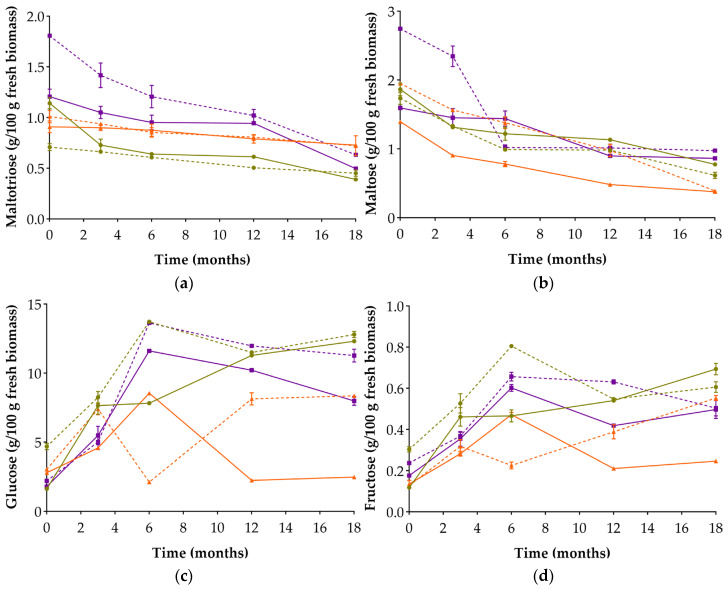
Concentration of reducing sugars in the six alternative legume-based misos over 18 months of fermentation: maltotriose (**a**); maltose (**b**); glucose (**c**); fructose (**d**). Values are expressed as mean ± standard deviation (*n* = 3). Circles: chickpea-based misos; triangles: lupin-based misos; squares: cowpea-based misos. Solid lines: 3% NaCl; dashed lines: 12% NaCl.

In terms of reducing sugars (Figure 3), maltotriose and maltose exhibited a general decreasing trend throughout fermentation. Among the six types of miso, LM3 displays a distinct profile: maltotriose remained stable, while maltose decreased gradually, suggesting limited starch hydrolysis—possibly due to matrix-related enzymatic inaccessibility [33]. In CoM12, higher early levels of both sugars may reflect lower enzymatic activity at the onset. Still, from T6 onwards, values in CoM3 and CoM12 converged, suggesting a compensatory increase in hydrolysis under high-salt conditions.

An opposite trend was observed for glucose and fructose, which increased as fermentation progressed. Although these monosaccharides are usually consumed at early stages, their accumulation can be explained by their continuous release through the hydrolysis of maltotriose, maltose, and more complex polysaccharides. Glucose remained consistently more abundant than fructose across all miso types, confirming its predominance as the main reducing sugar released during fermentation. Overall, miso produced with 12% NaCl tended to reach higher glucose and fructose concentrations than their 3% NaCl counterparts, in line with the results reported by Liu and co-authors [28]. CM3 exhibited the most pronounced increase in both sugars, and despite starting from the lowest initial levels, it gradually converged with CM12. This behaviour suggests that salt inhibition may have been progressively overcome in CM3, enabling greater enzymatic hydrolysis in the later stages of fermentation. Similar results were obtained by Lin et al. (2024) when studying the early stages of fermentation of high-salt (12%) and low salt (5%) rice koji soybean miso, reflected in the higher increase in reducing sugar observed for the low salt miso also attributed to a higher enzymatic activity under these conditions [44].

In contrast, LM3 ended with the lowest concentrations of both glucose and fructose and was the only miso in which glucose decreased between T0 and T18. A sharp drop in glucose at month 6 (73.6%) suggests intense metabolic activity at this stage. The persistently low sugar levels in LM3 may reflect a higher metabolic rate under reduced salt conditions, which are less inhibitory to hydrolytic enzymes, particularly in a matrix characterised by low starch and high protein content [33]. This pattern is also consistent with the lower pH values observed in this miso (see Section 3.2).

**Figure 4:**
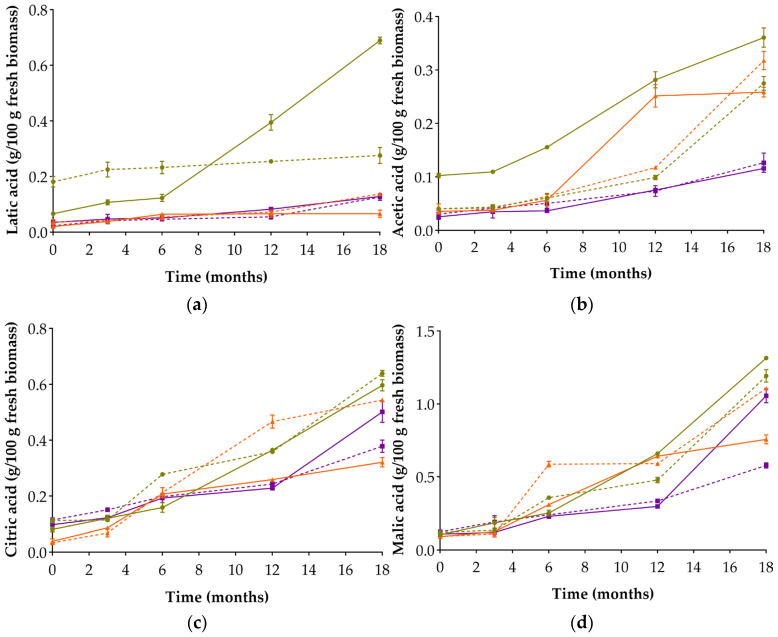
Concentration of organic acids in the six alternative legume-based misos over 18 months of fermentation: lactic acid (**a**); acetic acid (**b**); citric acid (**c**); malic acid (**d**). Circles: chickpea-based misos; triangles: lupin-based misos; squares: cowpea-based misos. Solid lines: 3% NaCl; dashed lines: 12% NaCl.

All miso types showed a progressive accumulation of organic acids during fermentation (Figure 4), although the patterns varied depending on the legume type and salt concentration.

CM3 also stood out for its fermentation acid profile, as it initially had high levels of lactic and acetic acids, and ultimately achieved the highest final concentrations—particularly of lactic acid. These findings suggest vigorous microbial activity [45], especially during the first six months, consistent with the sustained viability of LAB observed in CM3. A similar overall profile of viable cells was found in CoM12 (Table 2), which may reflect a matrix effect associated with cowpea. Conversely, CM12 followed a different pattern: although it had the highest initial lactic acid content, its concentration remained stable throughout fermentation.

In cowpea-based misos, the salt concentration appeared to have a limited effect on the accumulation of lactic and acetic acids—a pattern that remained consistent throughout fermentation. This behaviour may be attributed to the restricted solubilisation of proteins and phenolic compounds (Figure 2), which could have constrained microbial metabolism by reducing the availability of fermentable substrates [46,47].

The highest increase in acetic acid was recorded at T6 by LM3 (341.9%), coinciding with a pronounced decrease in glucose (73.6%). This observation is consistent with increased metabolic activity at this stage of the fermentation process.

Regarding citric and malic acids, chickpea-based misos consistently exhibited the highest concentrations of these non-fermentative acids (TCA cycle organic acids) at the end of fermentation. Apart from occurring naturally in the raw materials [48], these two organic acids can also be produced by *A. oryzae* [49]. The similar behaviour of CM3 and CM12—despite their different salt concentrations—suggests that acid accumulation was more strongly influenced by the chickpea matrix than by NaCl content. In cowpea-based misos, comparable levels of citric and malic acids were observed up to month 12, after which CoM3 showed a steeper increase, ending with higher final values than CoM12. This divergence may reflect enhanced enzymatic activity under low-salt conditions, which likely promoted greater matrix solubilisation, as supported by the higher TSS values and the sharper glucose depletion observed in CoM3 (Figure 2 and Figure 3).

LM12 exhibited greater accumulation of all organic acids than LM3—the only pair of misos in which the high-salt sample outperformed its counterpart. This miso presented higher final concentrations of glucose, fructose, and soluble phenolic compounds, suggesting that residual enzymatic activity—possibly retained from the koji—continued to support matrix degradation even after microbial decline [50,51].

### 3.5. Soybean-Based Misos

As previously mentioned in Section 2.4, the soybean-based misos were assessed at the start and end of fermentation, as shown in Table 3. These two time points allowed for an overall assessment of the impact of fermentation on their characteristics. Additionally, the final values enabled a direct comparison between the two soybean misos and the six innovative misos.

Regarding cell viability at T0, the soybean-based misos exhibited yeast and LAB counts comparable to those observed in the chickpea-based misos (Table 2). Specifically, SM3 and SM12 showed yeast counts of 4.255 and 4.064 log_10_ CFU/g, and LAB counts of 8.306 and 8.376 log_10_ CFU/g, respectively. As expected, no culturable microorganisms were detected in SM3 and SM12 after 18 months of fermentation.

Considering all endpoint measurements (Table 3), a similar evolution was observed between the six innovative misos and the soybean misos. For example, pH decreased, and TSS and soluble phenolic content increased in SM3 and SM12, consistent with the changes observed in the other misos. Furthermore, SM12 also ended with a higher pH than SM3. Despite these common trends, TSS values were generally lower and soluble phenolic compounds were higher in the traditional soybean miso, reflecting differences in the initial composition of the legumes.

Regarding sugars and organic acids, a similar tendency was observed for the increase in glucose and fructose, as well as in lactic, citric, and malic acids. Despite these similarities, a notable exception was observed: SM3 and SM12 exhibited an increase in maltotriose and maltose concentrations, and SM3 also showed a decrease in acetic acid levels—a change not observed in the other misos.

The distinct nutritional profile of cooked soybean—characterised by a higher lipid content, lower carbohydrate and starch levels, and relatively elevated concentrations of micronutrients (e.g., phosphorus, magnesium, and thiamine) compared to chickpea, lupin, and cowpea—may have influenced the fermentation environment [12,13,14,15,52,53], favouring the accumulation of fermentable sugars such as maltotriose and maltose. In addition, the lower salt concentration in SM3 compared with SM12 may have supported the activity of specific microbial populations and of enzymes. This higher activity could have increased the total concentration of reducing sugars, as previously observed during the early stages of fermentation [44,49], and affected acetic acid production [27].

Despite differences in the initial compositions of the legumes, the final characteristics of the six alternative legume-based misos were overall similar to those of the soybean-based misos, with the two chickpea misos showing the highest similarity to soybean misos.

## 4. Conclusions

Six innovative misos were produced from three different legumes (chickpea, lupin, and cowpea) traditionally consumed in Portugal but with low market value. These misos were prepared at two salt concentrations: 12%—a concentration standard for traditional soybean miso—and 3%, a low-salt concentration aligned with recent salt-reduction trends and consumers’ health concerns. The effects of legume and salt on the microbiological and physicochemical properties of long-term fermentation (18 months) were evaluated and compared with those of soybean misos with the same salt concentration.

The results showed that, in addition to fermentation time, the type of legume and the salt concentration influenced the performance of the enzymes and fermentative microorganisms present, and consequently the characteristics of the final product.

Chickpea-based misos exhibited the highest metabolic activity, which translated to the highest levels of soluble protein, phenolic compounds, reducing sugars, and organic acids. Conversely, cowpea-based misos showed more limited solute release, particularly in terms of soluble protein and phenolic compounds. In lupin-based misos, clear differences were observed between LM3 and LM12: despite the higher salt content, LM12 accumulated greater amounts of soluble phenolic compounds, reducing sugars, and organic acids.

When compared with soybean-based misos, alternative legume-based misos showed a similar fermentative evolution, suggesting that these legumes may represent suitable substrates for miso production. However, the number of studies exploring the production of these non-conventional misos is limited, and further research is needed to expand on the findings presented in this work, particularly regarding their overall quality of misos and the sensorial impact of the legumes and salt concentration.

The results obtained highlight the potential of these legumes as alternative substrates for miso production, supporting the development of sustainable, innovative, lower-salt products as a strategy to valorise low-market-value yet regionally relevant raw materials.

## Figures and Tables

**Table 1:** Nutritional composition of cooked legumes (per 100 g edible portion): soybean, chickpea, lupin, and cowpea.

Nutritional Component	Soybean	Chickpea	Lupin	Cowpea
Carbohydrates (g)	5.6 ^1^	16.7	7.2	18.1
Starch (g)	2	15.1	6.7	16.4
Fibre (g)	5.6	5.1	4.8	4.7
Protein (g)	12.5	8.4	16	8.8
Fat (g)	7.5	2.1	2.4	0.7
Vitamins				
Thiamine (mg)	0.3	0.1	0.12	0.19
Pyridoxine (mg)	0.16	0.14	0.06	0.1
Folate (µg)	64	54	84.5	210
Minerals				
Potassium (mg)	510	270	250	320
Calcium (mg)	82	46	48	21
Phosphorus (mg)	240	83	110	140
Magnesium (mg)	84	39	54	47
Iron (mg)	2.6	2.1	3.4	1.9
Zinc (mg)	1.4	1.2	1.4	1.1

^1^ All values refer to cooked, drained legumes. Data compiled from the Instituto Nacional de Saúde Doutor Ricardo Jorge (INSA), *Tabela da Composição de Alimentos* (2023) [17].

**Table 2:** Viable counts of culturable yeasts and lactic acid bacteria (LAB) in the six alternative legume-based misos over 270 days of fermentation.

Microorganism (log_10_ CFU/g Fresh Biomass)	Time (Day)	Alternative Legume-Based Misos					
		CM3	CM12	LM3	LM12	CoM3	CoM12
Yeasts	0	5.441 ± 0.009	4.090 ± 0.148	5.401 ± 0.064	5.744 ± 0.061	5.672 ± 0.052	5.280 ± 0.050
	15	3.597 ± 0.039	3.638 ± 0.136	7.180 ± 0.002	5.158 ± 0.090	6.436 ± 0.016	6.866 ± 0.004
	30	6.027 ± 0.038	3.380 ± 0.157	5.833 ± 0.247	3.423 ± 0.035	5.352 ± 0.008	3.744 ± 0.141
	60	NCC	NCC	3.866 ± 0.004	3.889 ± 0.184	3.498 ± 0.108	NCC
	90	NCC	NCC	NCC	4.922 ± 0.063	3.591 ± 0.016	NCC
	180	NCC	NCC	NCC	NCC	3.932 ± 0.098	NCC
	270	NCC	NCC	NCC	NCC	NCC	NCC
LAB	0	8.276 ± 0.092	5.623 ± 0.015	4.740 ± 0.090	5.009 ± 0.030	6.623 ± 0.001	6.208 ± 0.067
	15	6.613 ± 0.060	5.106 ± 0.060	4.607 ± 0.053	3.591 ± 0.111	4.968 ± 0.007	4.633 ± 0.072
	30	4.964 ± 0.013	3.884 ± 0.020	3.985 ± 0.244	3.854 ± 0.030	6.158 ± 0.001	5.055 ± 0.158
	60	5.602 ± 0.046	3.544 ± 0.035	3.591 ± 0.001	4.602 ± 0.015	3.942 ± 0.132	3.519 ± 0.037
	90	4.164 ± 0.248	NCC	NCC	4.460 ± 0.014	4.190 ± 0.129	3.672 ± 0.013
	180	5.276 ± 0.036	NCC	NCC	NCC	3.695 ± 0.043	3.380 ± 0.103
	270	NCC	NCC	NCC	NCC	NCC	NCC

NCC: Non-culturable cells. Values are expressed as mean ± standard deviation (*n* = 3). Miso codes are described in Section 2.4.

**Table 3:** Physicochemical parameters of alternative legume-based misos and soybean-based misos at the start and at the end of fermentation.

Parameters	Time (Months)	Alternative Legume-Based Misos						Soybean-Based Misos	
		CM3	CM12	LM3	LM12	CoM3	CoM12	SM3	SM12
pH	0	4.993 ± 0.015 ^d; (e)^	5.333 ± 0.025 ^c; (c)^	4.637 ± 0.021 ^h; (k)^	4.633 ± 0.012 ^h; (k)^	5.580 ± 0.010 ^a; (a)^	5.517 ± 0.006 ^b; (b)^	5.130 ± 0.032 ^(d)^	5.070 ± 0.020 ^(d)^
	18	4.797 ± 0.025 ^g; (j)^	4.830 ± 0.010 ^f; (i)^	4.223 ± 0.015 ^j; (m)^	4.467 ± 0.006 ^i; (l)^	4.837 ± 0.006 ^f; (i)^	4.973 ± 0.006 ^e; (f)^	4.877 ± 0.006 ^(h)^	4.937 ± 0.006 ^(g)^
TSS	0	14.667 ± 0.577 ^h; (h)^	12.000 ± 0.000 ^j; (j)^	9.000 ± 0.000 ^k; (l)^	13.333 ± 0.577 ^i; (i)^	21.000 ± 0.000 ^g; (g)^	12.000 ± 0.000 ^j; (j)^	10.000 ± 0.000 ^(k)^	25.667 ± 0.577 ^(f)^
	18	44.000 ± 0.000 ^c; (b)^	52.000 ± 0.577 ^a; (a)^	35.000 ± 0.000 ^f; (e)^	41.000 ± 0.000 ^d; (c)^	44.667 ± 0.577 ^b; (b)^	37.667 ± 0.577 ^e; (d)^	35.000 ± 0.000 ^(e)^	35.000 ± 1.000 ^(e)^
Soluble Protein (mg/100 g)	0	27.539 ± 0.463 ^cd; (c)^	25.243 ± 0.206 ^de; (d)^	9.172 ± 0.679 ^h; (h)^	9.495 ± 1.156 ^h; (h)^	17.522 ± 0.416 ^ef; (ef)^	16.638 ± 0.588 ^f; (f)^	21.332 ± 0.579 ^(e)^	25.328 ± 0.078 ^(d)^
	18	145.799 ± 8.988 ^ab; (a)^	148.690 ± 10.067 ^a; (a)^	46.473 ± 2.718 ^bc; (b)^	10.827 ± 0.368 ^g; (g)^	5.861 ± 0.793 ^i; (i)^	9.500 ± 1.767 ^h; (h)^	40.588 ± 4.847 ^(b)^	40.384 ± 3.915 ^(b)^
Soluble Phenolic Compounds (mg GAE/100 g)	0	5.024 ± 0.254 ^h; (k)^	4.424 ± 0.311 ^i; (l)^	3.803 ± 0.100 ^j; (m)^	5.830 ± 0.189 ^f; (i)^	5.634 ± 0.094 ^g; (j)^	3.585 ± 0.100 ^k; (n)^	4.196 ± 0.136 ^(l)^	9.797 ± 0.435 ^(h)^
	18	36.572 ± 0.671 ^a; (a)^	29.816 ± 0.906 ^b; (d)^	15.474 ± 1.016 ^d; (f)^	20.269 ± 0.987 ^c; (e)^	12.423 ± 1.199 ^e; (g)^	12.118 ± 1.310 ^e; (g)^	34.611 ± 0.400 ^(b)^	32.693 ± 1.953 ^(c)^
Reducing Sugar (g/100 g)									
Maltotriose	0	1.142 ± 0.055 ^b; (b)^	0.709 ± 0.034 ^d; (d)^	0.909 ± 0.058 ^c; (c)^	1.011 ± 0.062 ^c; (c)^	1.207 ± 0.073 ^ab; (ab)^	1.807 ± 0.016 ^a; (a)^	0.495 ± 0.030 ^(g)^	0.498 ± 0.014 ^(g)^
	18	0.391 ± 0.002 ^g; (h)^	0.452 ± 0.031 ^fg; (h)^	0.729 ± 0.005 ^d; (d)^	0.722 ± 0.099 ^de; (de)^	0.497 ± 0.010 ^f; (g)^	0.634 ± 0.007 ^e; (ef)^	0.994 ± 0.021 ^(c)^	0.610 ± 0.038 ^(f)^
Maltose	0	1.866 ± 0.093 ^b; (b)^	1.737 ± 0.088 ^c; (c)^	1.396 ± 0.004 ^e; (de)^	1.951 ± 0.017 ^b; (b)^	1.596 ± 0.007 ^d; (d)^	2.745 ± 0.024 ^a; (a)^	0.308 ± 0.006 ^(j)^	0.386 ± 0.013 ^(j)^
	18	0.775 ± 0.008 ^h; (g)^	0.615 ± 0.046 ^i; (g)^	0.378 ± 0.001 ^j; (i)^	0.390 ± 0.026 ^j; (i)^	0.863 ± 0.006 ^g; (f)^	0.977 ± 0.010 ^f; (e)^	0.881 ± 0.025 ^(f)^	0.425 ± 0.016 ^(h)^
Glucose	0	1.645 ± 0.085 ^l; (m)^	4.694 ± 0.223 ^f; (g)^	2.812 ± 0.148 ^h; (i)^	3.057 ± 0.027 ^g; (h)^	1.805 ± 0.011 ^k; (l)^	2.224 ± 0.017 ^j; (k)^	0.438 ± 0.038 ^(o)^	0.904 ± 0.061 ^(n)^
	18	12.322 ± 0.114 ^b; (b)^	12.806 ± 0.223 ^a; (a)^	2.495 ± 0.063 ^i; (j)^	8.366 ± 0.099 ^d; (d)^	7.989 ± 0.316 ^e; (e)^	11.282 ± 0.469 ^c; (c)^	12.992 ± 0.288 ^(a)^	6.922 ± 0.373 ^(f)^
Fructose	0	0.119 ± 0.007 ^h; (jk)^	0.304 ± 0.013 ^d; (fg)^	0.136 ± 0.018 ^g; (j)^	0.129 ± 0.004 ^gh; (i)^	0.177 ± 0.002 ^f; (i)^	0.238 ± 0.003 ^e; (hi)^	0.081 ± 0.07 ^(k)^	0.132 ± 0.021 ^(j)^
	18	0.694 ± 0.028 ^a; (a)^	0.606 ± 0.026 ^a; (bc)^	0.247 ± 0.009 ^e; (gh)^	0.553 ± 0.012 ^b; (cd)^	0.497 ± 0.043 ^c; (ef)^	0.504 ± 0.038 ^c; (de)^	0.652 ± 0.047 ^(ab)^	0.585 ± 0.050 ^(c)^
Organic Acids (g/100 g)									
Latic	0	0.067 ± 0.006 ^e; (f)^	0.182 ± 0.019 ^b; (cd)^	0.021 ± 0.003 ^g; (h)^	0.037 ± 0.009 ^f; (g)^	0.036 ± 0.007 ^f; (g)^	0.024 ± 0.003 ^g; (h)^	0.058 ± 0.006 ^(f)^	0.043 ± 0.002 ^(g)^
	18	0.690 ± 0.012 ^a; (f)^	0.276 ± 0.029 ^ab; (ab)^	0.067 ± 0.013 ^e; (f)^	0.138 ± 0.006 ^c; (de)^	0.129 ± 0.005 ^cd; (e)^	0.127 ± 0.012 ^d; (e)^	0.059 ± 0.011 ^(f)^	0.250 ± 0.008 ^(bc)^
Acetic	0	0.103 ± 0.004 ^d; (gh)^	0.041 ± 0.004 ^e; (i)^	0.036 ± 0.005 ^ef; (ij)^	0.041 ± 0.009 ^e; (i)^	0.026 ± 0.005 ^g; (k)^	0.031 ± 0.007 ^fg; (jk)^	0.159 ± 0.009 ^(e)^	0.114 ± 0.012 ^(fg)^
	18	0.361 ± 0.018 ^a; (a)^	0.275 ± 0.013 ^bc; (cd)^	0.259 ± 0.009 ^c; (de)^	0.318 ± 0.017 ^ab; (bc)^	0.116 ± 0.007 ^d; (fg)^	0.127 ± 0.018 ^d; (f)^	0.075 ± 0.003 ^(h)^	0.368 ± 0.034 ^(ab)^
Citric	0	0.082 ± 0.009 ^f; (i)^	0.113 ± 0.008 ^de; (gh)^	0.040 ± 0.009 ^g; (j)^	0.034 ± 0.002 ^g; (j)^	0.098 ± 0.014 ^e; (hi)^	0.115 ± 0.008 ^d; (g)^	0.030 ± 0.001 ^(k)^	0.033 ± 0.003 ^(j)^
	18	0.597 ± 0.020 ^a; (ab)^	0.640 ± 0.011 ^a; (a)^	0.322 ± 0.017 ^c; (ef)^	0.544 ± 0.006 ^b; (bc)^	0.502 ± 0.038 ^b; (cd)^	0.379 ± 0.022 ^c; (e)^	0.284 ± 0.011 ^b(f)^	0.484 ± 0.024 ^(d)^
Malic	0	0.104 ± 0.007 ^g; (hi)^	0.121 ± 0.010 ^fg; (g)^	0.092 ± 0.007 ^h; (ij)^	0.093 ± 0.001 ^h; (j)^	0.109 ± 0.016 ^g; (h)^	0.124 ± 0.006 ^f; (g)^	0.117 ± 0.011 ^(gh)^	0.127 ± 0.002 ^(g)^
	18	1.316 ± 0.008 ^a; (a)^	1.193 ± 0.042 ^ab; (ab)^	0.758 ± 0.030 ^de; (de)^	1.108 ± 0.010 ^bc; (b)^	1.057 ± 0.048 ^cd; (bc)^	0.580 ± 0.018 ^e; (ef)^	0.471 ± 0.007 ^(f)^	0.807 ± 0.025 ^(cd)^

Values are expressed as mean ± standard deviation (*n* = 3). Different letters within the same parameter indicate statistically significant differences (α = 0.05). Letters without parentheses refer to comparisons among alternative legume-based misos, whereas letters in parentheses refer to their comparisons with the soybean-based. Miso codes and fermentation times are detailed in Section 2.4.

## Data Availability

The original contributions presented in the study are included in the article, further inquiries can be directed to the corresponding author.
